# CSF-Glioma: A Causal Segmentation Framework for Accurate Grading and Subregion Identification of Gliomas

**DOI:** 10.3390/bioengineering10080887

**Published:** 2023-07-26

**Authors:** Yao Zheng, Dong Huang, Yuefei Feng, Xiaoshuo Hao, Yutao He, Yang Liu

**Affiliations:** 1 School of Biomedical Engineering, Air Force Medical University, No. 169 Changle West Road, Xi’an 710032, China; zhengyao0202@fmmu.edu.cn (Y.Z.); huangdong1007785@outlook.com (D.H.); deer_von@163.com (Y.F.); xiaoshuohao@fmmu.edu.cn (X.H.);; 2 Shaanxi Provincial Key Laboratory of Bioelectromagnetic Detection and Intelligent Perception, No. 169 Changle West Road, Xi’an 710032, China

**Keywords:** glioma grading, deep learning, explanations

## Abstract

Deep networks have shown strong performance in glioma grading; however, interpreting their decisions remains challenging due to glioma heterogeneity. To address these challenges, the proposed solution is the Causal Segmentation Framework (CSF). This framework aims to accurately predict high- and low-grade gliomas while simultaneously highlighting key subregions. Our framework utilizes a shrinkage segmentation method to identify subregions containing essential decision information. Moreover, we introduce a glioma grading module that combines deep learning and traditional approaches for precise grading. Our proposed model achieves the best performance among all models, with an AUC of 96.14%, an F1 score of 93.74%, an accuracy of 91.04%, a sensitivity of 91.83%, and a specificity of 88.88%. Additionally, our model exhibits efficient resource utilization, completing predictions within 2.31s and occupying only 0.12 GB of memory during the test phase. Furthermore, our approach provides clear and specific visualizations of key subregions, surpassing other methods in terms of interpretability. In conclusion, the Causal Segmentation Framework (CSF) demonstrates its effectiveness at accurately predicting glioma grades and identifying key subregions. The inclusion of causality in the CSF model enhances the reliability and accuracy of preoperative decision-making for gliomas. The interpretable results provided by the CSF model can assist clinicians in their assessment and treatment planning.

## 1. Introduction

Glioma grading is a crucial aspect of diagnosing and treating brain tumors. Accurate grading provides essential information about tumor aggressiveness and helps guide treatment decisions for patients [1,2]. Deep learning models have shown impressive performance in glioma grading by utilizing medical image data [3,4]. However, the lack of interpretability of these models poses a significant challenge for their practical implementation, which is vital for gaining trust and acceptance among medical professionals. Understanding and explaining the decisions made by deep learning models are crucial for their successful integration into the diagnostic workflow [5,6]. Consequently, there is an increasing demand for interpretable machine learning algorithms that provide not only accurate predictions but also understandable explanations.

Currently, several interpretable methods for deep learning models exist, primarily based on class activation mapping (CAM) [7,8,9,10]. These methods rely on assumptions and correlations to explain the model’s decisions, which are considered reasonable due to their apparent validity. However, current interpretable methods can be seen as passive explanations [11], as their main objective is to explain the decisions made by pre-existing models. These methods suffer from some limitations:

1. Failure to establish causality: Most interpretability methods often explain the model’s decisions based on activated regions of a feature map from a trained model. There is no specific causal relationship between these regions and model decisions. Without clear causality, it is difficult to know whether the black-box model obtains information from regions beyond the identified critical region.
2. Rough explanations: The explanations provided are often insufficient in resolution to accurately identify key regions. This is often due to multiple downsampling and the low-resolution design of the model itself, which usually does not take into account the quality of the visual interpretation.

In order to address this issue, the Causal Segmentation Framework (CSF) has been developed. This framework employs an end-to-end approach to segment gliomas and identify key subregions. It extracts features from these subregions to predict high and low grades of glioma. The CSF incorporates a key region identification module that adheres to the principle of causality. Through the shrinkage segmentation process, features are extracted to enhance key region identification and improve classification performance while maintaining causality. Additionally, our glioma grading module consists of two paths, deep features and traditional features, thereby ensuring accurate grading.

Unlike existing interpretability methods that provide rough and passive explanations, our algorithm actively identifies and localizes the specific regions within the tumor that influence the grading outcome. By incorporating the principle of causality, it establishes a stronger link between the identified subregions and the model’s decisions. This enhances the interpretability of the algorithm and provides valuable insights for medical professionals to understand the grading process.

Furthermore, our algorithm integrates both deep features extracted from medical images and traditional features commonly used in glioma grading. This combination ensures the accuracy and reliability of the grading results, and it allows for a more comprehensive assessment of tumor characteristics.

Our contributions are as follows:

1. An end-to-end network is proposed in this scientific study to accurately predict the grades of glioma while also providing outputs of key subregions for prediction.
2. A subregion identification process is introduced in this study, aligning with the principle of causality. The utilization of shrinkage segmentation enables the identification of subregions that possess significant decision-making information.
3. In this study, a glioma grading module is developed that integrates deep features and traditional features, facilitating the accurate grading of gliomas.

In summary, our proposed algorithm addresses the need for interpretable machine learning models in glioma grading. By leveraging causal segmentation and integrating deep and traditional features, it provides accurate predictions along with transparent and comprehensive explanations. The algorithm developed in this research exhibits the potential to facilitate the integration of deep learning models into clinical practice, thereby contributing to enhanced patient care.

## 2. Related Work

### 2.1. Glioma Grading

The grading of gliomas based on medical images has been extensively studied. Traditional methods typically involve feature extraction, target region selection, and the establishment of a discrimination model [12]. Feature extraction techniques often include texture features, the co-occurrence matrix, and wavelet transform, among others. The gray-level co-occurrence matrix (GLCM) is considered an effective feature for capturing the spatial relationship of pixels [13]. Discriminant models used in traditional approaches include the SVM [14], decision tree [15], LASSO [16], and various other methods. These traditional processes rely on hand-designed features, which can ensure model stability.

In recent years, convolutional neural networks (CNNs) have gradually replaced traditional methods due to their excellent performance. Various types of CNN architectures, such as the simple CNN [17], ResNet [18], Inception [19], and RNN [20], have achieved remarkable results in glioma classification tasks. However, interpreting these results can be challenging, and identifying the key regions responsible for predictions would undoubtedly aid in the clinical application of these models.

### 2.2. Method of Explanations

As mentioned above, identifying key subregions is crucial for the clinical application of the model. Currently, class activation mapping (CAM) [7] can be considered as the pioneering approach in this field. CAM re-trains the model by introducing global average pooling in order to obtain the weights of feature maps. To avoid changing the model structure, gradient-weighted class activation mapping (Grad-CAM) [21] determines the weight of feature maps by calculating the gradient weight of each map. Grad-CAM++ [21] claims to provide better results by using element-wise feature weights.

The abovementioned methods are widely used, and they are considered state-of-the-art for interpreting models. However, these methods often associate key subregions with activation weights, and this lacks a causal understanding. To address this issue, an active search method is proposed. This method involves segmenting the subregions based on the predictions of the model and ensuring that these subregions make a full contribution to the overall prediction. By doing so, we establish a causal relationship between the identified subregions and the model’s predictions.

## 3. Methods

### 3.1. Problem Definition

The glioma grading dataset is denoted as $D={\left\{X_{i},y_{i}\right\}}_{i=1}^{n}$. Specifically, in this study, $X_{i}\in R^{4\times H\times W}$ denotes the *i*-th image with 4 modalities where image slice size H×W is 240×240 and $y_{i}\in \left\{0:low\;grade,1:high\;grade\right\}$ is the *i*-th label. The proposed model aims to predict $y_{i}$ for $X_{i}$, and provide the key decision subregion $K_{i}\in R^{1\times H\times W}$. The key decision subregion represents the specific region within the image that entirely influences the model’s prediction.

### 3.2. Architecture

**Figure 1:**
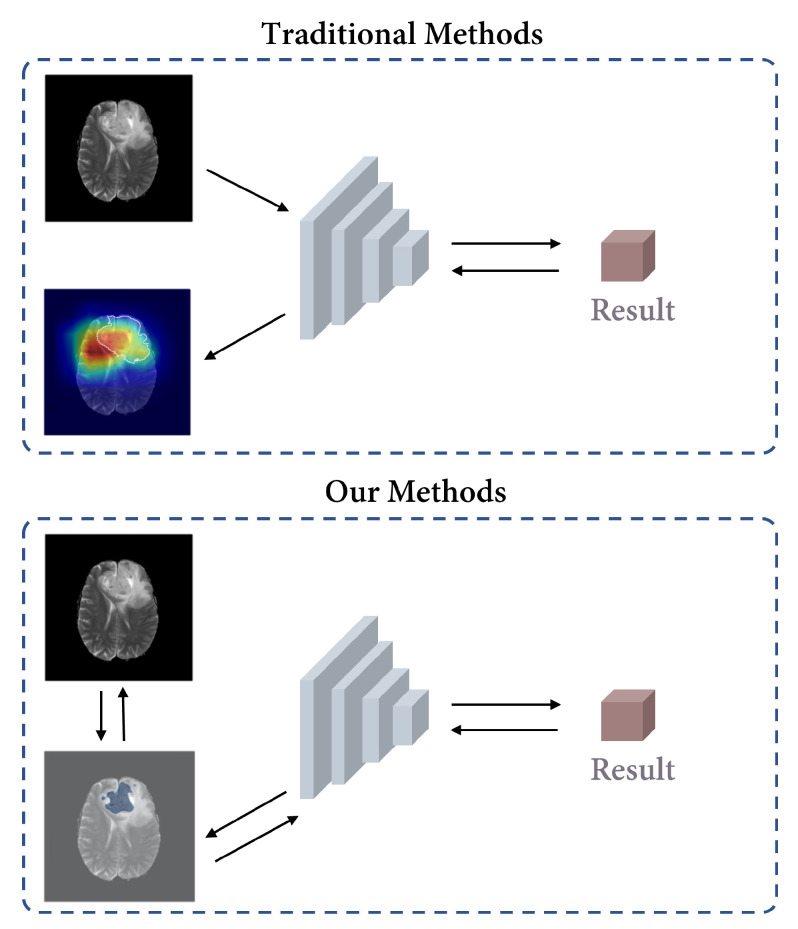
The figure provided illustrates our motivation for proposing a causal approach in our method. Traditional interpretable methods identify key regions based on assumptions, but they may overlook the contribution of other regions. Different colors in a heatmap represent varying levels of attention. In this example, the transition from red to blue indicates a descending order of attention. In contrast, our model actively segments subregions and integrates them into the decision network, ensuring that all decision-related information originates from the identified key subregions, establishing strong causality between regions and the model’s decisions. The blue color in our method represents the location of key subregions.

**Figure 2:**
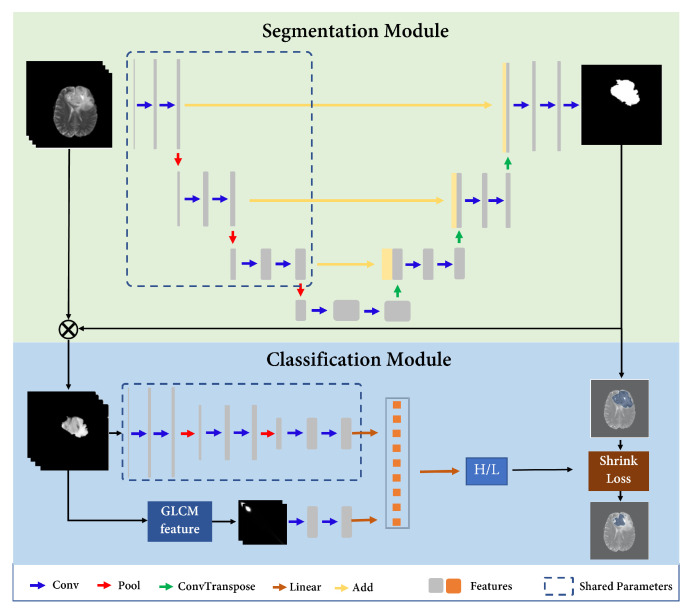
The overall architecture of the proposed Causal Segmentation Framework (CSF).

As illustrated in Figure 1, our model is based on the idea of ensuring that all the information utilized for the decision-making process originates from the identified key subregions, thereby establishing a stronger causality between the regions and the model’s decisions. Figure 2 shows the overall architecture of the proposed Causal Segmentation Framework (CSF), which is composed of two main parts and one training strategy. **Segmentation Module:** a UNet-like model [22] is employed for glioma segmentation. **Classification Module:** the classification module consists of a deep learning branch and a traditional branch. The deep learning branch shares the same parameters with the encoder of segmentation module. The traditional branch extracts gray-level co-occurrence matrix (GLCM) features to ensure the stability of prediction. **Training Strategy:** a three-step training strategy, including a segmentation step, a classification step, and a shrink step, is proposed to complete model training.

### 3.3. Segmentation Module

In this module, a UNet-like model is used to complete glioma segmentation. Specifically, for input $X\in R^{4\times H\times W}$, it is first operated by a convolution with a kernel size of 3 × 3 to generate feature maps $F_{0}\in R^{32\times H\times W}$. Then, four dual convolution operations and three downsampling operations are applied to calculate depth features. For each level, the dual-convolution operation contains two convolution blocks; the first block is used to double the channel numbers of the feature maps, and the second block is used to further extract features in the same channel numbers. The downsampling operation is set as a max-pooling operation. Each convolution block consists of a BatchNorm2d, a 3 × 3 convolution layer, and a Leaky ReLU function. For λ-th level feature map $F_{\lambda }$, the encoding process is denoted as:

(1) $$F_{\lambda }=E\left(\theta_{E},X,\lambda \right)=\left\{\begin{array}{cc} DualConv\left(Conv_{3\times 3}\left(X_{i}\right)\right)\in R^{C_{1}\times H_{1}\times W_{1}}, & \lambda =1\\ DualConv\left(Maxpool\left(F_{\lambda -1}\right)\right)\in R^{C_{\lambda }\times H_{\lambda }\times W_{\lambda }}, & \lambda =\left\{2,3,4\right\} \end{array}\right.$$

where $\theta_{E}$ represents the parameters of the corresponding encoder, $Conv_{3\times 3}$ denotes the initial 3 × 3 convolution, DualConv denotes the dual-convolution operations, Maxpool denotes max-pooling operations, and λ is the level of the encoder; $C_{\lambda }=16\times 2^{\lambda }$, $H_{\lambda }=H/2^{\lambda -1},W_{\lambda }=W/2^{\lambda -1}$ are the number of channels, height, and width.

The feature maps calculated by the encoder are of rich depth and spatial information. As opposed to the coding process of the encoder, three upsampling operations and three dual-convolution operations are used to decode the feature maps. The upsampling operation is a transposed convolution with a stride of two. The dual convolution operation is used to reduce channel numbers by half. Finally, a 1 × 1 convolution followed by a sigmoid activation function is applied to generate the segmentation results, and the process is denoted as:

(2) $${\tilde{F}}_{\lambda }=D\left(\theta_{D},F_{\lambda },\lambda \right)=\left\{\begin{array}{cc} F_{\lambda }\in R^{C_{4}\times H_{4}\times W_{4}}, & \lambda =4\\ DualConv\left(ConCat\left(TransConv\left({\tilde{F}}_{\lambda +1}\right),F_{\lambda }\right)\right)\in R^{C_{\lambda }\times H_{\lambda }\times W_{\lambda }}, & \lambda =\left\{3,2,1\right\}\\ Sigmoid\left(Conv_{1\times 1}\left({\tilde{F}}_{\lambda +1}\right)\right)\in R^{1\times H\times W}, & \lambda =0 \end{array}\right.$$

where $\theta_{D}$ represents the parameters of the corresponding decoder, $Conv_{1\times 1}$ denotes the 1 × 1 convolution, TransConv is the transposed convolution, and ConCat denotes the operation of stacking two matrices in the channel dimension. Therefore, ${\tilde{F}}_{4}$ is the segmentation result, and threshold 0.5 is used to generate mask:

(3) $$Mask_{i,j}=\left\{\begin{array}{cc} 1, & {\tilde{F}}_{4,i,j}\geq 0.5\\ 0, & {\tilde{F}}_{4,i,j}<0.5 \end{array}\right.$$

### 3.4. Classification Module

After the segmentation module, the segmented image $\tilde{X}\in R^{4\times H\times W}$ is calculated by Mask:

(4) $${\tilde{X}}_{i,j}^{c}=X_{i,j}^{c}\times Mask_{i,j}$$

where ${\tilde{X}}_{i,j}$ represents the gray value of the pixel in the *i*-th row and *j*-th column of the image.

In order to achieve an accurate and stable prediction of the glioma grade, we propose a two-branch classification module that extracts both deep and traditional features. For the $\tilde{X}$, the encoder of segmentation is used to extract deep learning features, and the process is denoted below:

(5) $$F_{deep}=Linear\left(E\left(\theta_{E},\tilde{X},\lambda \right)\right)$$

where Linear denotes the linear layer, whose input is the encoded feature map, and the output is $F_{deep}\in R^{10\times 1}$, and λ is set as 3.

For the traditional feature branch, the GLCM is applied to extract traditional features. In past research, the GLCM [23] has proven to be a good texture feature that has achieved promising performance. Consequently, the feature was employed to improve the model’s performance for subregions of varying sizes.

Let the gray level be *L*, and limit the pixel values in the image to the range [0,L−1]. Assume *d* represents the distance between pixels, and θ represents the direction between the pixels. Then, the GLCM can be represented as:

(6) $$GLCM_{\theta ,d}^{c}\left(i,j\right)=\frac{1}{N_{\theta ,d}}\sum_{h=1}^{H}\sum_{w=1}^{W}\delta \left({\tilde{X}}_{h,w}^{c},i\right)\delta \left({\tilde{X}}_{h+p,w+l}^{c},j\right)$$

where $N_{\theta ,d}$ represents the number of pixel pairs in direction θ and distance *d*, δ(x,y) represents the Kronecker delta symbol, which is 1 when x=y and 0 otherwise. *H* and *W* represent the number of rows and columns in the image, respectively, and *p* and *l* represent the offset in direction θ. *L* is 8, θ is set as $\left\{0^{{}^{\circ}},45^{{}^{\circ}},90^{{}^{\circ}}\right\}$, and *d* is 1. Then, $F_{traditon}\in R^{10\times 1}$ is calculated by the convolution operation and linear layer:

(7) $$F_{traditon}=Linear\left(Conv_{3\times 3}\left(Conv_{3\times 3}\left(GLCM_{0^{{}^{\circ}},1},GLCM_{45^{{}^{\circ}},1},GLCM_{90^{{}^{\circ}},1}\right)\right)\right)$$

Finally, deep learning features and traditional features are used together to predict the score *S* for glioma grade:

(8) $$S=Sigmiod(Linear\left(ConCat\left(F_{deep},F_{traditon}\right)\right))$$

where Sigmiod denotes the sigmoid activation function, and ConCat denotes the operation of stacking two matrices in the channel dimension.

### 3.5. Training Strategy

To output key subregions based on accurate prediction of glioma grade, a three-step training strategy was proposed.

1. In the initial step, the training of the segmentation module was accomplished using the dice loss function.
  (9) $$Dice\_loss=1-\frac{2\times \sum_{i=1}^{N}p_{i}\times g_{i}}{\sum_{i=1}^{N}p_{i}^{2}+\sum_{i=1}^{N}g_{i}^{2}}$$
  where *N* is the number of pixels in the predicted result, $p_{i}$ represents the predicted value of the *i*-th pixel by the model, and $g_{i}$ represents the true label value of the *i*-th pixel.
2. In the second step, a mixture loss was utilized to train the classification module, ensuring the preservation of segmentation accuracy.
  (10) $$Mix\_loss=\frac{1}{2}\left(Dice\_loss+CE\_loss\right)$$
  where CE_loss can be denoted as:
  (11) $$CE\_loss=-\frac{1}{N}\sum_{i=1}^{N}y_{i}\times log\left(s_{i}\right)+\left(1-y_{i}\right)\times log\left(1-s_{i}\right)$$
  where *N* is the number of samples, $y_{i}$ represents the true label value of the *i*-th sample (either 0 or 1), and $s_{i}$ represents the predicted probability value of the *i*-th sample.
3. In the third step, a shrinkage loss was proposed to complete the shrinkage of the segmentation Mask and thus output the key subregion *K*:
  (12) $$Shrinkage\_loss=\frac{1}{2+\alpha }\left(CE\_loss+INC\_loss+\alpha \times Shrinkage\_penalty\right)$$
  where α is weight coefficient for Shrinkage_penalty, and INC_loss can be denoted as:
  (13) $$INC\_loss=\frac{\left|Mask-G\right|}{\left|G\right|+1}$$
  where Mask represent the set of predicted mask of segmentation module and *G* is the ground truth label of tumor. The $\left|\cdot \right|$ is the cardinality of the corresponding set. Shrinkage_penalty can be denoted as:
  (14) $$Shrinkage\_penalty=\frac{\left|Mask\right|}{\left|G\right|+1}$$
  where α is set to 0.05 in this study.

The trained model can simultaneously predict the grading score *S* and output the corresponding key subregion mask *K*.

## 4. Setup and Results of Experiments

In the experimental section, a comprehensive analysis and comparison of the models’ performance is provided. To demonstrate the effectiveness of the model components and training strategy, an ablation experiment was conducted. This involved using an incomplete model to verify the superiority of the missing part, thereby highlighting the importance and contribution of each component. Subsequently, the scalability and sensitivity of the model were also specifically analyzed to prove the model stability. In these experiments, hyperparameters including dataset size, learning rate, and optimizer were varied in order to see if the model performance changed substantially. Furthermore, the proposed model was compared with existing methods in several aspects, including multiple classification metrics, runtime, and memory. Finally, to highlight the superiority of the proposed model in the task of finding critical regions, multiple existing visualization methods were used for comparison. We also used several examples to show specific visual effects. We explain the key points in as much detail as possible. If not specified, it is usually the default setting for the deep learning framework, PyTorch [24].

### 4.1. Data

All images were obtained from multimodal brain tumor segmentation (BraTS 2019) [25]. The dataset includes 335 × 4 images from 335 subjects with four modalities: T1-weighted (T1), T2-weighted (T2), postcontrast T1-weighted (T1Gd), and T2 fluid attenuated inversion recovery (FLAIR). In this study, for each subject, the slice with max tumor is selected in our dataset $D={\left\{X_{i},y_{i}\right\}}_{i=1}^{335}$. The image size H×W is 240 × 240, and the labels contain 259 high-grade and 76 low-grade images.

### 4.2. Training Details

The PyTorch [24] deep learning framework was used for the model, running on an NVIDIA V100 GPU. To train the model, the optimizer was set to SGD. The learning rate was set to 0.002, while the batch size was 12. In order to increase data diversity, random flipping, stretching, and augmenting were used for data augmentation to better train the models.

We initialized our model parameters using the default initialization method in PyTorch, which utilizes Xavier uniform initialization. Xavier uniform initialization ensures that the weights of the neural network are initialized within an appropriate range, alleviating the problems of gradient vanishing or exploding and also enhancing network stability and convergence.

### 4.3. Evaluation Metrics

To evaluate the classification performance of the models, classification accuracy (ACC), F1 score (F1), sensitivity (SEN), specificity (SPE), and the area under the curve (AUC) of the tumor grade were all used. The data were divided 8:2 into a training set and a test set; the model was trained on the training set, and the performance was tested on the test set.

### 4.4. Ablation Study

**Figure 3:**
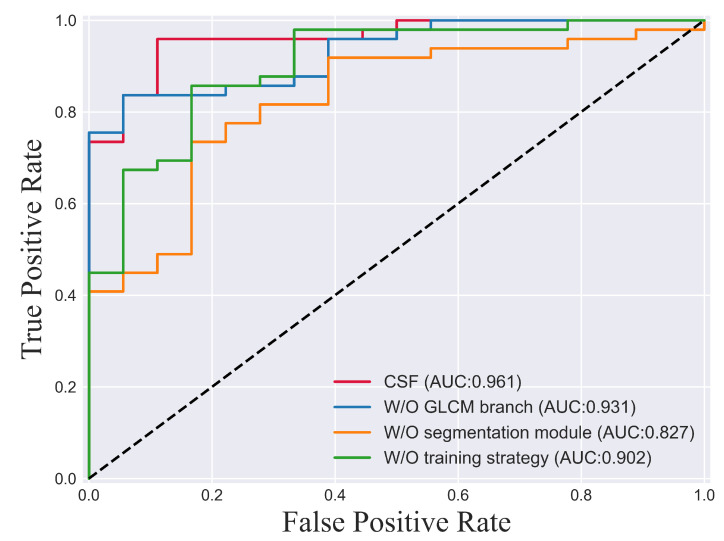
ROC curve of the ablation experiment.

To verify the effectiveness of the proposed modules or strategies (the two-branch classification module, the three-step training strategy, and the segmentation module), ablation experiments were performed.In this study, the degradation of the model’s classification performance was specifically tested by removing each structure individually (the two-branch classification module was changed to the single deep learning classification module, the three-step training strategy was changed to one-step common training, and the segmentation module was removed). This allowed us to assess the impact of each structure on the overall classification performance. The results are shown in Table 1 and Figure 3.

#### 4.4.1. Effectiveness of the Two-Branch Classification Module

To verify the effectiveness of incorporating traditional feature branches in improving the classification performance of the model, an experiment was conducted. In this experiment, the gray-level co-occurrence matrix (GLCM) branch was removed, and the results were compared with the original model. The purpose was to assess the impact of the GLCM branch on the overall classification performance. The evaluation showed a decrease in almost all of the performance metrics, with the AUC dropping from 96.14% to 93.08%. This indicates that the addition of the traditional feature branch effectively enhances the model’s classification performance.

Furthermore, when examining the sensitivity and the specificity of the results, we observed that a single deep learning branch may lead to imbalanced sensitivity and specificity. Specifically, the model exhibited excessively high sensitivity and low specificity. This imbalance can negatively impact the model’s generalization performance as it becomes more prone to false positives. Therefore, incorporating multiple branches helps to address this issue and provides a more balanced prediction.

#### 4.4.2. Effectiveness of the Training Strategy

To validate the effectiveness of the proposed three-step training strategy, an experiment was conducted where the network was trained directly using only the cross-entropy loss as the training objective. This experiment aimed to compare the performance of the network trained with the three-step strategy against the network trained solely with the cross-entropy loss. The results demonstrated a noticeable decrease in performance, with the AUC dropping by 6 percentage points to 90.24%. This highlights the significance of our training strategy in aiding the model in identifying key subregions compared to simple direct training.

#### 4.4.3. Effectiveness of the Segmentation Module

The segmentation module employed in our framework plays a crucial role in guiding the model to focus on relevant subregions, contributing to the improved generalization performance. To evaluate the impact of the segmentation module on glioma grading, it was removed from the framework, and the network was trained without it. This allowed for an assessment of how the absence of the segmentation module affected the overall performance of the grading process. The results showed a significant decrease in model performance, with the AUC dropping to 82.65%. This suggests that without the segmentation module, the model may incorrectly shrink to irrelevant subregions, leading to inadequate generalization performance. Hence, the segmentation module proves effective at facilitating glioma grading.

### 4.5. Analysis of Scalability and Sensitivity

To better analyze the proposed model, we comprehensively analyzed the scalability and the sensitivity of the model [26,27].

**Figure 4:**
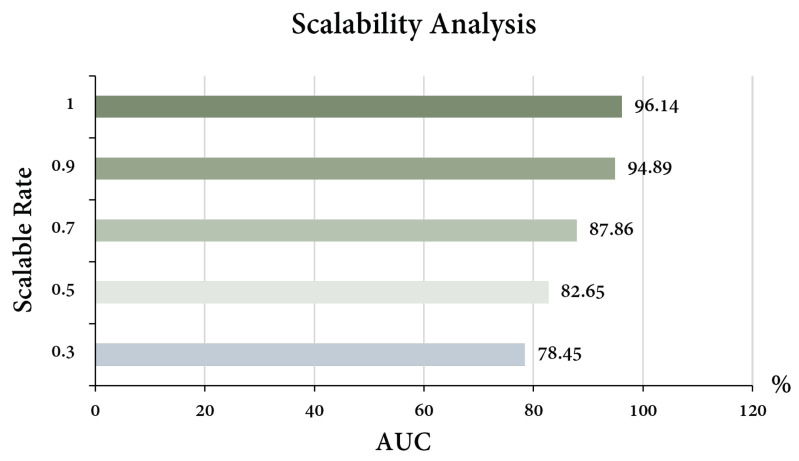
The results of scalability analysis. Different colors, representing different AUC, were provided merely to enhance visual contrast.

The training set sizes were reduced in order to the original 0.3, 0.5, 0.7, and 0.9, and the corresponding AUC was calculated on the same test set to observe the scalability of the model. The results are shown in Figure 4: the larger the training set, the better the performance of the model. However, even at the size of 0.3, the model still achieved an AUC of 78.45%, which proved that the scalability of the model itself was good.

**Figure 5:**
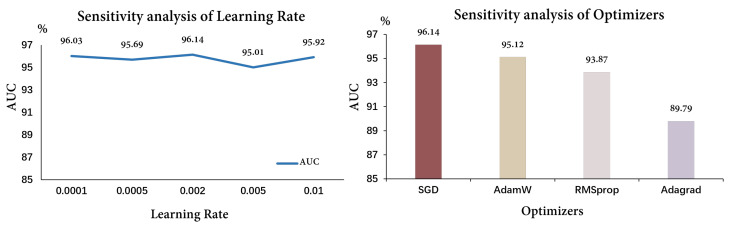
The results of sensitivity analysis. For sensitivity analysis of optimizers, different colors, representing different AUC, were provided merely to enhance visual contrast.

Secondly, to analyze the model’s sensitivity, we experimented with different optimizers and learning rates, which are crucial hyperparameters. In this study, we implemented several optimizers, including SGD, AdamW, RMSprop, and Adagrad, using the PyTorch framework [24]. These optimizers were employed to optimize the training process of our model. Additionally, we tested learning rates of 0.0001, 0.0005, 0.002, 0.005, and 0.01. The corresponding results are illustrated in Figure 5. Through comparisons using different settings, we found that the model is more sensitive to the choice of optimizer, and that SGD had a better performance, likely due to its smoother optimization process.

### 4.6. Comparison with Strong Baseline Classification Methods

To demonstrate the better classification performance of our framework, we compared the proposed model with some baseline classification models, including ResNet [28], SEResNet [29], EfficientNet [30], DenseNet [31], MobileNetV3 [32], and VGG [33]. The results are shown in Table 2. For each model, the DeLong test [34] was used to test whether the proposed model AUC was significantly better than the baseline model.

Overall, our proposed model achieved the best performance among all of the models, with an AUC of 96.14%, an F1 score of 93.74%, an accuracy of 91.04%, a sensitivity of 91.83%, and a specificity of 88.88%, which proved the superiority of our proposed framework.

In addition to this, the running time and the memory occupied by all of the models on the test set were calculated. Our proposed model takes 2.31 s and occupies 0.12 GB of memory, which means that the proposed model can complete the prediction with small resource occupation. Our model utilizes parameter reuse to reduce the memory footprint. However, in order to ensure causal inference during prediction, information needs to pass through relevant modules, which unavoidably increases the processing time. Nevertheless, the additional time required falls within an acceptable range.

One point worth mentioning is that our model seems to be somewhat lower in terms of specificity. This is because the identification of a high level is often only necessary to identify the key high-level subregions, while the identification of low level usually requires consideration of the whole tumor. Therefore, it is often difficult for low-level predictions to search for their subregions, and we need to carefully adjust the model parameters to balance specificity and subregion searching.

### 4.7. Comparison with Baseline Visualization Methods

**Figure 6:**
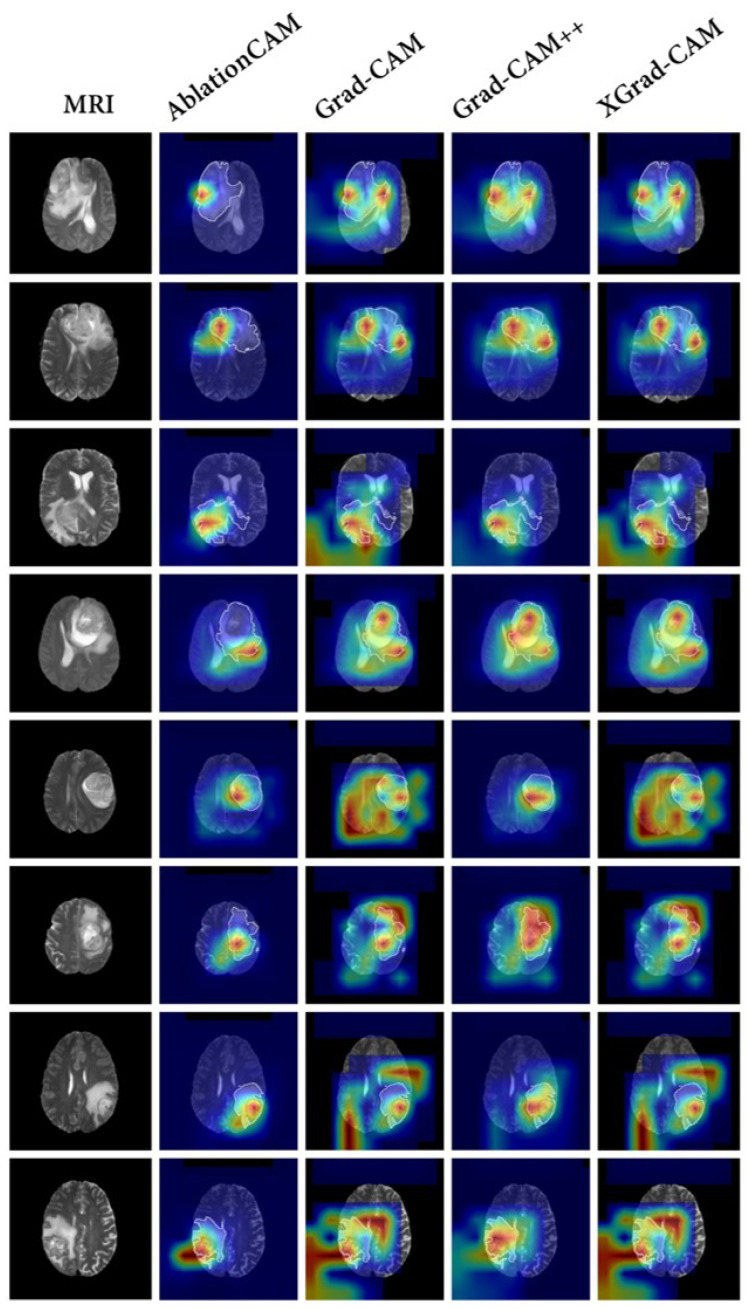
The results of baseline visualization methods. In the traditional methods, AblationCAM [35], Grad-CAM [21], Grad-CAM++ [36], and XGrad-CAM [37] are used to explain the trained SEResNet [29]. Specifically, The first column shows the T2 fluid attenuated inversion recovery (FLAIR) MRI. Columns 2–5 show the visual explanations for AblationCAM [35], Grad-CAM [21], Grad-CAM++ [36], and XGrad-CAM [37], respectively. Different colors in a heatmap represent varying levels of attention. In these examples, the transition from red to blue indicates a descending order of attention.

To compare our proposed interpretable method with conventional methods, we conducted a comparison using the baseline model SEResNet, which exhibited the best classification performance. We selected AblationCAM [35], Grad-CAM [21], Grad-CAM++ [36], and XGrad-CAM [37] as the baseline visualization methods to explain the model’s decision-making process. The results are shown in Figure 6.

In general, the results of the baseline models exhibited a diffuse distribution pattern. Among all of the baseline methods, AblationCAM [35] performed the best and was able to focus on the tumor region more accurately. However, it still exhibited some false attention, such as focusing on the black background. This false attention is often problematic in interpretation methods, since the model does not utilize any information from the black background that would be beneficial for classification.

**Figure 7:**
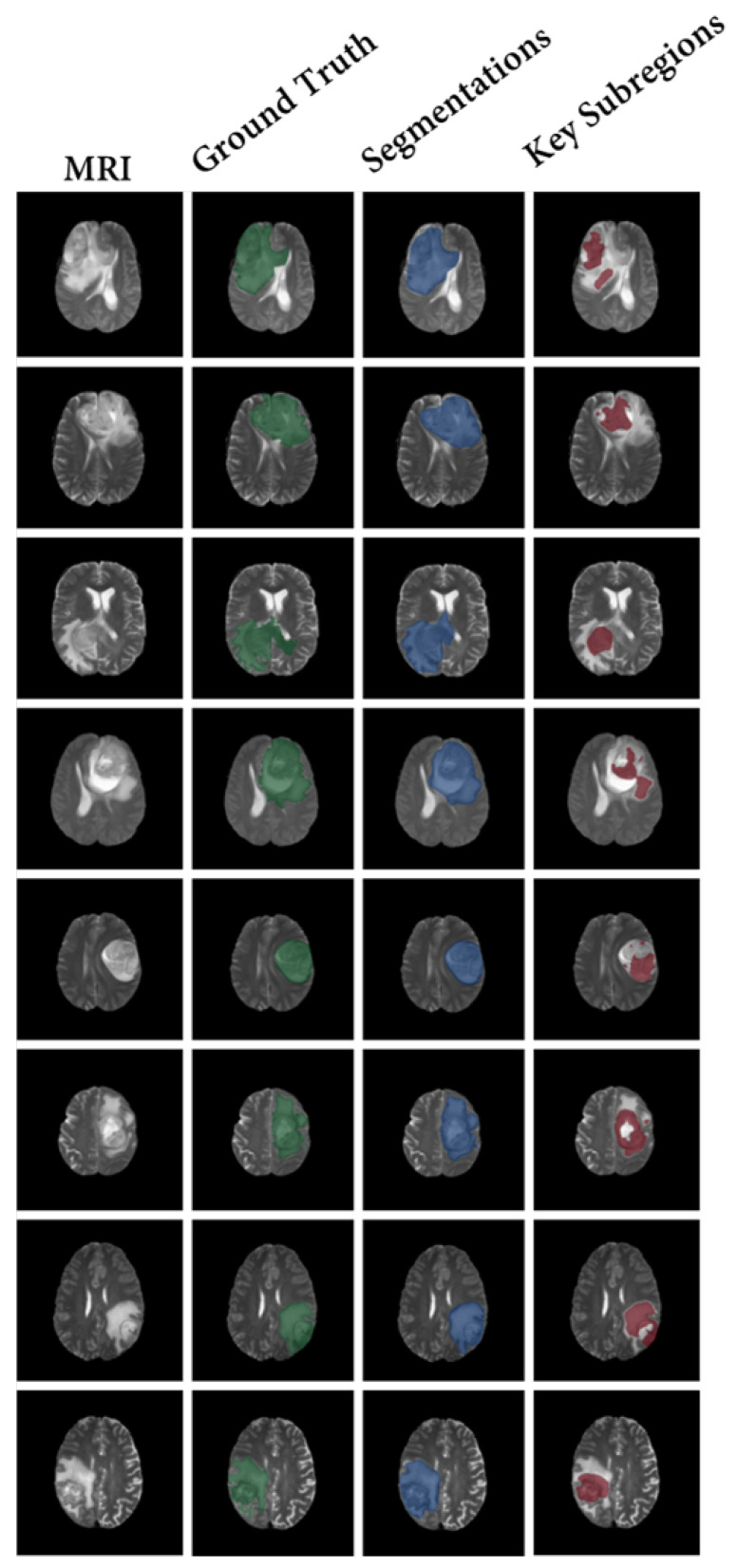
The results of our framework (CSF). This result shows the decision-making process of the model, that is, how the model shrinks to key subregions. Specifically, the first column shows the T2 fluid attenuated inversion recovery (FLAIR) MRI. Columns 2–4 show the ground truth of tumor segmentation (Green), the segmentation results of the model (Blue), and the shrinkage results of the model (Red), respectively.

In contrast, the proposed CSF demonstrates the search process of key subregions, as shown in Figure 7. After training the segmentation results of our model using the proposed strategy, the segmented subregions are reduced to the key subregions.

Our method provides clear and specific visualization of the key subregions, which is significantly better than the vague explanations provided by other methods. It is important to note that, due to the causal aspect of our model design, these subregions inherently contain the complete information required for making predictions.

Furthermore, the shrinkage strategy employed in our model helps to focus on the correct areas and reduce misclassified attention. This contributes to the overall improvement of classification performance.

## 5. Discussion

In recent years, deep-learning-based preoperative grading of glioma images has shown promising performance. However, the interpretation of the decision-making process in glioma grading remains a challenging task. Gliomas are heterogeneous tumors, and different regions within the tumor may have varying influence on the prediction. Therefore, identifying the key regions that contribute to the decision-making process could be valuable for clinical applications.

Traditional methods often rely on certain explanations and correlations to identify key subregions. However, these methods may have limitations. The interpretations provided by these methods may not be causal, meaning there is no guarantee that the model focuses only on the regions deemed significant. Additionally, the low resolution of some model designs may lead to rough explanations.

The proposed Causal Segmentation Framework (CSF) is an end-to-end network that accurately predicts glioma grades while also providing the output of the key subregions for the prediction. The CSF incorporates a shrinkage segmentation method to identify subregions with sufficient decision information, as well as a glioma grading module that combines both deep learning and traditional features for accurate grading.

The complexity of our proposed model lies in achieving a balance between resource utilization and prediction performance. We have addressed this challenge by adopting parameter reuse techniques, effectively reducing memory requirements without compromising prediction accuracy. Additionally, our model’s architecture has been optimized to ensure causal inference, albeit at the expense of slightly increased processing time. By carefully managing these factors, we strike a favorable balance between model complexity and performance. Our approach maximizes performance while minimizing the demand for computational resources, making it practical and efficient for real-world applications.

In addition to achieving the best performance among all the models, with an AUC of 96.14%, an F1 score of 93.74%, an accuracy of 91.04%, a sensitivity of 91.83%, and a specificity of 88.88%, our proposed model also exhibits efficient resource utilization. The running time and memory occupied by all of the models on the test set were calculated, and our proposed model completes the prediction within 2.31 s, occupying only 0.12 GB of memory. Our approach provides clear and specific visualizations of the key subregions, which outperforms other methods in terms of interpretability. This important distinction is highlighted because the inherent causal relationships in our model’s design ensure that these subregions inherently contain the complete information necessary for making predictions. These findings further support the superiority of our proposed framework in terms of performance, resource efficiency, and clear explanations.

Experimental results demonstrate that our model achieves superior performance and visualization results, which can greatly benefit the clinical application of the model.

Compared to other models, our proposed model has several advantages:

Firstly, our model achieves better classification performance. For metrics such as AUC, F1 score, accuracy, and sensitivity, the model outperforms baseline classification models, highlighting the superiority of our framework.

Secondly, our interpretable method surpasses conventional approaches in terms of accuracy and reliability. The key subregions identified by our model are more distinct and specific, which is crucial for clinical application.

Thirdly, our model is designed with causality, ensuring that the identified subregions are truly the key regions that drive the predictions. This represents a significant improvement over passive explanations that lack causality.

In terms of future work, there are plans to enhance the performance of the model by incorporating more advanced deep learning techniques. Additionally, exploring the applicability of the model to other medical imaging tasks is an objective of this research.

However, our study also has several limitations. Despite selecting the largest tumor slice for experimentation, it is important to note that it may not capture all the spatial information. Exploring the stability of a three-dimensional active search model remains an important area for future investigation. Second, this model requires careful selection of training strategies, which can be complex. Simplifying this process and improving the robustness of training are goals for future exploration.

## 6. Conclusions

In this paper, the Causal Segmentation Framework (CSF) is proposed for the accurate prediction of glioma grades and the identification of key subregions. The CSF incorporates a shrinkage segmentation method and a glioma grading module with both deep learning and traditional features. Our proposed model outperforms all other models, achieving a superior performance with an AUC of 96.14%, an F1 score of 93.74%, an accuracy of 91.04%, a sensitivity of 91.83%, and a specificity of 88.88%. Moreover, our model demonstrates efficient resource utilization, making predictions in just 2.31 s and utilizing a mere 0.12 GB of memory during the test phase. Additionally, our approach excels in interpretability, offering clear and detailed visualizations of key subregions, surpassing existing methods in this aspect. Experimental results demonstrate that the CSF model achieves better classification performance and visualization capabilities compared to other models. The CSF model is designed with causality, improving the accuracy and reliability of preoperative decision-making for gliomas. Its ability to provide interpretable results can benefit clinicians in their assessment and treatment planning.

## Figures and Tables

**Table 1:** Results of the ablation experiment (%).

Model	F1	ACC	SEN	SPE	AUC
W/O GLCM branch	87.49	79.10	**99.99**	22.22	93.08
W/O training strategy	88.88	82.08	97.95	38.88	90.24
W/O segmentation module	71.60	65.67	59.18	83.33	82.65
Ours (CSF)	**93.74**	**91.04**	91.83	**88.88**	**96.14**

**Table 2:** Comparison with strong baseline classification methods (%).

Model	F1	ACC	SEN	SPE	AUC	Runtime	Memory
ResNet [28]	89.32	83.58	93.87	55.55	91.95 *	0.57 s	0.32 GB
DenseNet121 [31]	85.55	80.59	75.51	**94.44**	94.33	0.69 s	0.11 GB
SEResNet [29]	90.72	86.56	89.79	77.78	95.80	0.61 s	0.33 GB
EfficientNet [30]	86.31	80.59	83.67	72.22	86.50 *	0.65 s	0.12 GB
MobileNetV3 [32]	89.13	85.07	83.67	88.88	93.42 *	0.55 s	0.02 GB
VGG [33]	91.30	88.05	85.71	94.44	89.17 *	0.73 s	2.01 GB
Ours (CSF)	**93.74**	**91.04**	**91.83**	88.88	**96.14**	2.31 s	0.12 GB

* Significantly worse (p<0.05) than our proposed method.

## Data Availability

All data used in this article are derived from public datasets, and proper citations have been included as required.

## Abbreviations

The following abbreviations are used in this manuscript:

GLCM	Gray-level co-occurrence matrix
CAM	Class activation mapping
CSF	Causal Segmentation Framework
CNN	Convolutional neural network
ACC	Accuracy
F1	F1 score
SEN	Sensitivity
SPE	Specificity
AUC	Area under the curve
